# Supplementary material for: Evolution of the Chinese guarantee network under financial crisis and stimulus program
Source: Nat Commun. 2020 Jun 1;11:2693. doi: 10.1038/s41467-020-16535-8 (PMC7264362; doi:10.1038/s41467-020-16535-8)
Supplement: Supplementary file 1 — Supplementary Information [file 41467_2020_16535_MOESM1_ESM.pdf]

Supplementary Information

Evolution of the Chinese Guarantee Network under Financial Crisis and Stimulus Program

Wang et al.

This PDF file includes:

Supplementary Figure 1
Supplementary Figure 2
Supplementary Table 1
Supplementary Note 1: Related Work
Supplementary Note 2: Significance test with directed configuration model
Supplementary Note 3: Analysis of weighted guarantee network
Supplementary Note 4: Simulation with various attack strategies
Supplementary References

Supplementary Figure 1

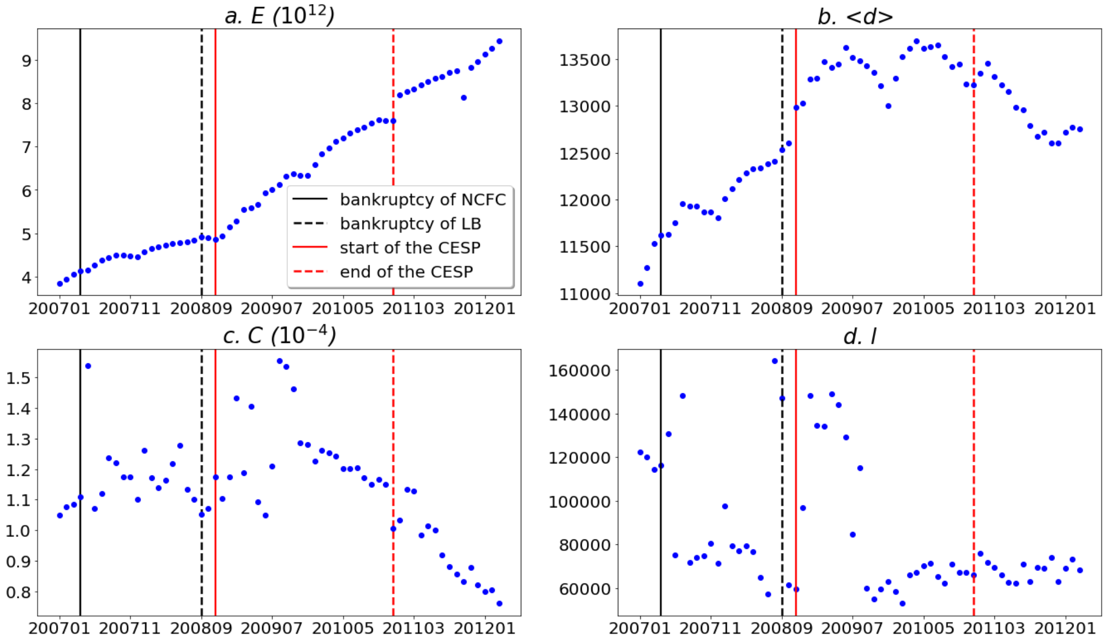

Supplementary Fig. 1. Dynamics of the topological properties of the weighted guarantee network. (a) *E*: network size. (b) <*d*>: average degree of weighted guarantee network. (c) *C*: average clustering coefficient of weighted guarantee network. (d) *l*: average directed shorted path length within strongly connected giant component of weighted guarantee network.

Supplementary Figure 2

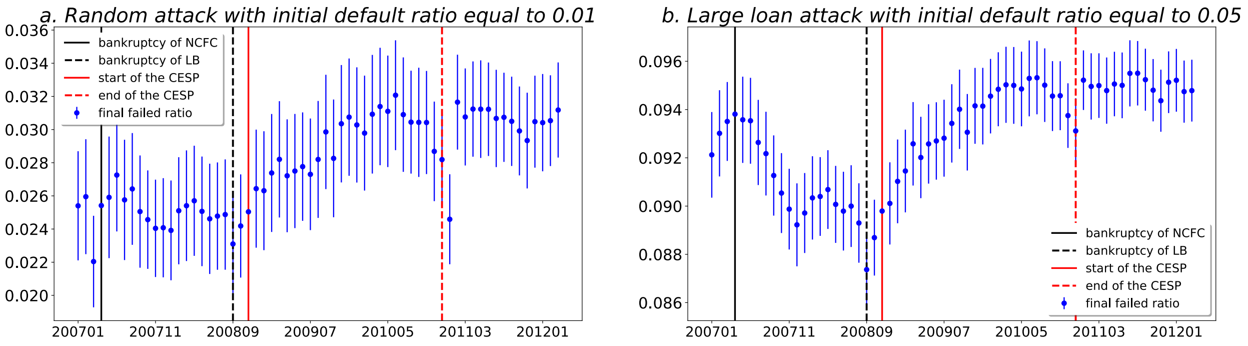

Supplementary Figure 2. Simulation results of random attack with initial default ratio η equal to 0.01, and targeted attack with initial default ratio η equal to 0.05. The error bar represents one standard deviation.

Supplementary Table 1

Supplementary Table 1. Summary of the topological and financial properties of the guarantee network in three phases.

Measure	Phase 1 (04/07-11/08)		Phase 2 (12/08-12/10)		Phase 3 (01/11-03/12)		Whole period (01/07-03/12)	
	Mean	SD	Mean	SD	Mean	SD	Mean	SD
N	38343.62	818.265	48062.35	6425.67	67130.87	4167.02	48975.6	12323.52
E	36891.925	739.893	46981.12	6372.16	65825.87	4202.5	47769.52	12329.30
**<*d*>**	0.96	0.0019132	0.98	0.01	0.98	0.01	0.97	0.01
$\boldsymbol{\lambda_{in}}$	3.30	0.145	3.23	0.07	3.23	0.04	3.29	0.18
$\boldsymbol{\lambda_{out}}$	2.30	0.03	2.74	0.15	2.76	0.05	2.58	0.25
Δ	2.51E-05	6.10E-07	2.07E-05	2.80E-06	1.47E-05	8.92E-07	2.1 E-05	4.7 E-06
C (%)	0.97	0.04	1.49	0.152	1.41	0.05	1.23	0.257
WCC	10583.955	180.67	13279.54	1726.90	18994.6	1656.6	13720.36	3560.46
WCC (%)	27.57	0.45	27.66	0.60	28.35	0.71	27.99	0.76
WCN	12301.575	155.62	15286.62	1897.23	21372.87	1698.35	15669.06	3871.35
SCC	211.15	35.95	325.32	33.45	446.73	29.98	307.69	103.65
SCC (%)	0.56	0.08	0.67	0.06	0.66	0.02	0.62	0.11
SCN	34850.05	1030.70	44392.24	5554.45	61542.2	3889.55	44861.80	11103.26
r (%)	13.84	0.19	14.80	0.41	14.36	0.35	14.37	0.51
2-node (%)	3.56	0.10	3.82	0.17	3.56	0.25	3.65	0.22
3-node (%)	1.15	0.09	1.34	0.11	1.34	0.05	1.26	0.14
l (weakly)	14.52	0.285	18.07	1.86	18.40	0.42	16.93	2.13
l (strongly)	10.54	1.75	10.10	0.78	10.24	0.48	10.12	1.33
AA (million)	109986.975	104.18	2446.23	60.65	2597.22	61.85	2387.25	194.79
AL (million)	20279.09	10.03	452.61	12.61	407.71	9.94	423.40	28.48
ALR (%)	60	1	61	8	62	4	61	1
ARL (%)	4.67	0.08	3.73	0.53	2.74	0.08	3.95	0.09

N: number of nodes; *E*: number of edges; <*d*>: average out/in-degree; λ_{in}: power-law index of in-degree distribution; λ_{out}: power-law index of out-degree distribution; Δ: density; *C*: average clustering coefficient (directed); *WCC*: size of the largest weakly connected component (giant component); *WCC (%)*: ratio of weakly connected giant component (%); *WCN*: weakly connected component number; *SCC*: size of the largest strongly connected component (giant component); *SCC (%)*: ratio of strongly connected giant component (%); *SCN*: strongly connected component number; *r*: reciprocity; *2-node (%)*: ratio of isolated 2-node reciprocal component (%); *3-node(%)*: ratio of fully connected three nodes (%); *l (weakly)*: average shortest path length in weakly connected giant component; *l (strongly)*: average shortest path length in strongly connected giant component; *AA (million)*: average assets of firms ; *AL (million)*: average loans of firms; *ALR (%)*: average leverage ratio (total liabilities/total assets) of firms. *ARL (%)*: average ratio of listed firms (number of listed firm / number of all firms). *Mean* and *SD* are the mean value and standard deviation of different properties within Phase *i* (i=1,2,3).

Supplementary Note 1: Related Work

Network science perspective

In the past decade, complex network has emerged as an effective tool to model and study real-world complex systems of different domains, including social networks, biological networks, Internet, collaboration networks, citation networks etc [1]. Recently, there has been a growing interest in applying network science to solve economic and financial problems [2,3] [4]. For example, network analysis has already been applied extensively to investigating the global banking system [5], international financial network [6], and interlocking boards of directors [7-9]. These studies focused on characterizing the topological properties of the financial and business networks, and using these properties to interpret the individual and organizational behaviors.

Over the past decades, the links between topological characteristics and robustness of network have been extensively studied [10]. We classify these studies into three major types. The first one is the analysis of the "robust-yet-fragile" property of networks [11]. Within a certain range, connections serve as a shock absorber, and risk sharing prevails, so connectivity engenders robustness. However, beyond a certain range, interconnections serve as shock-amplifiers, as losses cascade. The second one is the "fat-tailed distribution" of networks. In particular, fat-tailed distributions have been shown to be more robust to random disturbances, but more susceptible to targeted attacks. Because a targeted attack on a hub node can bring most part of the system into a crisis, whereas random attacks are most likely to fall on the periphery. The third one is the well-known "small world" property of networks [12]. In general, firms in the guarantee networks tend to form local clustering or neighborhoods. So local disturbances tend to have higher chance to result in global effects [13]. Financial safety is a main concern of governments and banks. However, empirical research on the robustness and resilience of large-scale financial networks is still rare. Therefore, this study is a timely contribution to the quantitative understanding of the association between the topologies and the robustness of guarantee network.

Guarantee networks

In a guarantee relationship, the guarantor needs to assume the debt obligation of a debtor if she defaults [14-16]. Therefore, such relationships represent the financial responsibilities and venues for potential risk contagion between firms. The firms and their guarantee relationships form a complex guarantee network.

Guarantees make it easier for firms to acquire loans from banks, and could reduce the risk for banks [17-19]. Existing literature mainly focused on small-scale guarantee networks. The factors determining if Chinese listed firms' would participated in the guarantee network have been investigated [20]. Various methods have been adopted to assess the credit risk of individual firms in small-scale guarantee networks. For example, a contagion model was constructed to explore the risk among 13 SMEs [21]. Another similar contagion model was used to model the critical conditions triggering infection [22]. With the loan guarantee data of 2007 from one Chinese commercial bank, a k-shell decomposition-based method, Netrating, was developed to assess risk of firms [23]. With a ten-year loan guarantee records from another major Chinese commercial bank, a boosting model [24] and visual analytics approaches have been used to assess firms' credit risk [25]. An empirical

study from Chinese listed showed the role of guarantor didn't have a significant effect on the firm's default risk[26]. A study from Korean chaebol affiliates' loan guarantees identified the positive and negative effects of loan guarantee [17].

Given the recent progress in the analysis of guarantee network, research on the risk of a comprehensive nationwide guarantee network is yet to come. In addition, there is no research on the influence of economic situation and national economic policies on the topological structure of guarantee network. The 2007-2008 financial crisis and the subsequent Chinese economic stimulus program are two perfect natural experiments to investigate the influence of economic situation and national economic policies on the structure of guarantee network, as well as the associated contagion risk in the system.

Supplementary Note 2: Significance Test with Directed Configuration Model

First, we use the Directed Configuration Model (DCM) to generate 10,000 random networks. The nodes in each generated network should have the same in- and out-degree as the real guarantee network.

Second, we calculate the number of sub-pattern i in the real and random networks.

Third, calculate the Z-score of sub-pattern i.

$$Z_i = \frac{N_i(G^*) - < N_i(G) >}{\delta(N_i(G))},$$

where $N_i(G^*)$ is the occurrence of sub-pattern i in real network G^*, $< N_i(G) >$ is the expected occurrence of 10000 DCM ensemble graph, and $\delta(N_i(G))$ is the standard deviation of a series of $N_i(G)$. Thus, the larger the Z_i-score, the more statistically significant the sub-pattern i.

Supplementary Note 3: Analysis of Weighted Guarantee Network

To check the robustness of the findings above, we constructed weighted guarantee networks with the amount of loans as the weight on edges, and did the same analyses. Here, the weight on each edge is set to be the amount of the loan guarantee, which indicates the trust between the two firms, as well as the risk associated with this guarantee relationship. Note that the values of the many topological properties (such as density and reciprocity) are not affected by the weight of edges. Supplementary Figure 1 presents the dynamic of four topological properties, network size, average in-/out-degree, average clustering coefficient and average shortest path length, which are re-calculated with the edge weight. In particular, the network size is the sum of all edge weights. The in-/out-degree of a node is the sum of the edge weights for incoming/outcoming edges incident to the node. The clustering coefficient is defined as the geometric average of the weights of the subgraph edge [27]. The average directed shortest path of weighted network is calculated in the strongly connected giant component of network with guarantee loan of firms as edge weight. Please refer to [28] for detailed definitions. We found that the patterns were consistent with the main results found by unweighted network. The general trend and change points were similar to their counterparts shown in Figures 1 and 2 in the main manuscript.

Supplementary Note 4: Simulation with Various Attack Strategies

To check the robustness of the simulation results, we also performed sensitivity analysis with a smaller value of the initial default rate $\eta = 0.01$, and with a targeted attack strategy, which prioritized the attack on the firms with large total loan amount. Supplementary Figure 2 shows the final failed ratio of firms. The results are highly consistent with Fig. 5.

Supplementary References

1 Newman, M. Networks. Oxford university press, (2018).

2 Alshamsi, A., Pinheiro, F. L. & Hidalgo, C. A. Optimal diversification strategies in the networks of related products and of related research areas. Nature Communications 9, 1-7 (2018).

3 Fagiolo, G., Squartini, T. & Garlaschelli, D. Null models of economic networks: the case of the world trade web. Journal of Economic Interaction and Coordination 8, 75-107 (2013).

4 Stavroglou, S. K., Pantelous, A. A., Stanley, H. E. & Zuev, K. M. Hidden interactions in financial markets. Proceedings of the National Academy of Sciences 116, 10646-10651 (2019).

5 Minoiu, C. & Reyes, J. A. A network analysis of global banking: 1978–2010. Journal of Financial Stability 9, 168-184 (2013).

6 Chinazzi, M., Fagiolo, G., Reyes, J. A. & Schiavo, S. Post-mortem examination of the international financial network. Journal of Economic Dynamics and Control 37, 1692-1713 (2013).

7 Conyon, M. J. & Muldoon, M. R. The small world network structure of boards of directors. Available at SSRN: https://ssrn.com/abstract=546963 (2004).

8 Davis, G. F., Yoo, M. & Baker, W. E. The small world of the American corporate elite, 1982-2001. Strategic Organization 1, 301-326 (2003).

9 Robins, G. & Alexander, M. Small worlds among interlocking directors: Network structure and distance in bipartite graphs. Computational & Mathematical Organization Theory 10, 69-94 (2004).

10 Dey, A. K., Gel, Y. R. & Poor, H. V. What network motifs tell us about resilience and reliability of complex networks. Proceedings of the National Academy of Sciences 116, 19368-19373 (2019).

11 Watts, D. J. A simple model of global cascades on random networks. Proceedings of the National Academy of Sciences 99, 5766-5771 (2002).

12 Watts, D. J. & Strogatz, S. H. Collective dynamics of ‘small-world’networks. Nature 393, 440-442 (1998).

13 Haldane, A. G. Rethinking the financial network. Speech delivered at the Financial Student Association, Amsterdam, April 28 (2009).

14 De Aghion, B. A. On the design of a credit agreement with peer monitoring. Journal of Development Economics 60, 79-104 (1999).

15 Columba, F., Gambacorta, L. & Mistrulli, P. E. Mutual Guarantee institutions and small business finance. Journal of Financial Stability 6, 45-54 (2010).

16 Zecchini, S. & Ventura, M. The impact of public guarantees on credit to SMEs. Small Business Economics 32, 191-206 (2009).

17 Doh, T. & Ryu, K. Analysis of loan guarantees among the Korean Chaebol affiliates. International Economic Journal 18, 161-178 (2004).
18 Jiangpo, P. The Credit Guarantee System of Medium-and-small Enterprises Based on Mutual Guarantee. Journal of Financial Research 2, 75-82 (2008).
19 Cowan, K., Drexler, A. & Yañez, Á. The effect of credit guarantees on credit availability and delinquency rates. Journal of Banking & Finance 59, 98-110 (2015).
20 Jian, M. & Xu, M. Determinants of the guarantee circles: The case of Chinese listed firms. Pacific-Basin Finance Journal 20, 78-100 (2012).
21 Leng, A., Xing, G. & Fan, W. Credit risk transfer in SME loan guarantee networks. Journal of Systems Science and Complexity, 1-13 (2017).
22 Zhang, Z.-x., Li, P.-x. & Guo, J.-e. The infection mechanism of the guarantee chain crisis. Systems Engineering 4 (2012).
23 Meng, X., Tong, Y., Liu, X., Chen, Y. & Tan, S. Netrating: Credit risk evaluation for loan guarantee chain in china. Pacific-Asia Workshop on Intelligence and Security Informatics, Springer 99-108 (2017).
24 Niu, Z. et al. A hybrid approach for risk assessment of loan guarantee network. arXiv preprint arXiv:1702.04642 (2017).
25 Cheng, D. Visual analytics for loan guarantee network risk management. arXiv preprint arXiv:1705.02937 (2017).
26 Leng, A., Zhang, J. & Xing, G. Loan guarantees and guarantor default risk: Evidence from china. Available at SSRN 2536695 (2014).
27 Saramäki, J., Kivelä, M., Onnela, J.-P., Kaski, K. & Kertesz, J. Generalizations of the clustering coefficient to weighted complex networks. Physical Review E 75, 027105 (2007).
28 Newman, M. E. Analysis of weighted networks. Physical Review E 70, 056131 (2004).
