# Supplementary material for: Evolution of the Chinese guarantee network under financial crisis and stimulus program
Source: Nat Commun. 2020 Jun 1;11:2693. doi: 10.1038/s41467-020-16535-8 (PMC7264362; doi:10.1038/s41467-020-16535-8)
Supplement: Supplementary file 3 — Reporting Summary [file 41467_2020_16535_MOESM3_ESM.pdf]

nature research

Corresponding author(s): Qingpeng Zhang, Xiaoguang Yang

Last updated by author(s): May 6, 2020

Reporting Summary

Nature Research wishes to improve the reproducibility of the work that we publish. This form provides structure for consistency and transparency in reporting. For further information on Nature Research policies, see Authors & Referees and the Editorial Policy Checklist.

Statistics

For all statistical analyses, confirm that the following items are present in the figure legend, table legend, main text, or Methods section.

n/a	Confirmed	
☐	☒	The exact sample size (*n*) for each experimental group/condition, given as a discrete number and unit of measurement
☐	☒	A statement on whether measurements were taken from distinct samples or whether the same sample was measured repeatedly
☐	☒	The statistical test(s) used AND whether they are one- or two-sided<br>*Only common tests should be described solely by name; describe more complex techniques in the Methods section.*
☐	☒	A description of all covariates tested
☐	☒	A description of any assumptions or corrections, such as tests of normality and adjustment for multiple comparisons
☐	☒	A full description of the statistical parameters including central tendency (e.g. means) or other basic estimates (e.g. regression coefficient) AND variation (e.g. standard deviation) or associated estimates of uncertainty (e.g. confidence intervals)
☐	☒	For null hypothesis testing, the test statistic (e.g. *F*, *t*, *r*) with confidence intervals, effect sizes, degrees of freedom and *P* value noted<br>*Give P values as exact values whenever suitable.*
☒	☐	For Bayesian analysis, information on the choice of priors and Markov chain Monte Carlo settings
☒	☐	For hierarchical and complex designs, identification of the appropriate level for tests and full reporting of outcomes
☒	☐	Estimates of effect sizes (e.g. Cohen's *d*, Pearson's *r*), indicating how they were calculated

Our web collection on statistics for biologists contains articles on many of the points above.

Software and code

Policy information about availability of computer code

Data collection	The data was provided by one of China's major regulatory bodies ranging 01/2007-03/2012. The data is not sharable due to the restrictions and consent we signed with the regulatory body.
Data analysis	The codes for our study are open source at Github: https://github.com/ElfLu/Chinese-credit-network-.git

For manuscripts utilizing custom algorithms or software that are central to the research but not yet described in published literature, software must be made available to editors/reviewers. We strongly encourage code deposition in a community repository (e.g. GitHub). See the Nature Research guidelines for submitting code & software for further information.

Data

Policy information about availability of data

All manuscripts must include a data availability statement. This statement should provide the following information, where applicable:

- Accession codes, unique identifiers, or web links for publicly available datasets
- A list of figures that have associated raw data
- A description of any restrictions on data availability

The dataset used in the paper is confidential and only available to reader upon request. The source data underlying all figures, tables are provided as a Source Data file.

Field-specific reporting

Please select the one below that is the best fit for your research. If you are not sure, read the appropriate sections before making your selection.

☐ Life sciences ☒ Behavioural & social sciences ☐ Ecological, evolutionary & environmental sciences

For a reference copy of the document with all sections, see nature.com/documents/nr-reporting-summary-flat.pdf

Behavioural & social sciences study design

All studies must disclose on these points even when the disclosure is negative.

Study description	We analyzed the topological properties of the guarantee relationships between Chinese firms during 01/2007-03/2012.
Research sample	In this research, we acquire a comprehensive nationwide guarantee relationship dataset. The data is hosted and provided by the Academy of Mathematics and Systems Science of Chinese Academy of Sciences. The data spans from January 2007 to March 2012, and contains the monthly information for all loans extended to the client firms, which have a credit line above 50 million RMBs. These loan guarantee data are from all the 19 major banks of China, including the largest five state-owned commercial banks, twelve joint equity commercial banks, and two major policy banks (Import-Export Banks of China and China Development Bank), which account for nearly 80% total loans in China. Totally, there are about 0.3 million guarantee relationships during 01/2007-03/2012, which covers around 0.1 million borrowing firms located in 30 province-level regions of China.
Sampling strategy	This is a full dataset from the regulatory body.
Data collection	The data was provided by the regulatory body.
Timing	The full data was collected on 01/01/2013.
Data exclusions	No data was excluded.
Non-participation	This is a secondary analysis of the guarantee relationships between firms. Non-participation is not applicable.
Randomization	We analyzed the full data without randomization.

Reporting for specific materials, systems and methods

We require information from authors about some types of materials, experimental systems and methods used in many studies. Here, indicate whether each material, system or method listed is relevant to your study. If you are not sure if a list item applies to your research, read the appropriate section before selecting a response.

Materials & experimental systems

n/a	Involved in the study	
☒	☐	Antibodies
☒	☐	Eukaryotic cell lines
☒	☐	Palaeontology
☒	☐	Animals and other organisms
☒	☐	Human research participants
☒	☐	Clinical data

Methods

n/a	Involved in the study	
☒	☐	ChIP-seq
☒	☐	Flow cytometry
☒	☐	MRI-based neuroimaging
